# Temporary Proximal Tibial Epiphysiodesis for Correction of Leg Length Discrepancy in Children—Should Proximal Fibular Epiphysiodesis Be Performed Concomitantly?

**DOI:** 10.3390/jcm10061245

**Published:** 2021-03-17

**Authors:** Adrien Frommer, Maike Niemann, Georg Gosheger, Maria Eveslage, Gregor Toporowski, Andrea Laufer, Thomas Ackmann, Robert Roedl, Bjoern Vogt

**Affiliations:** 1 Pediatric Orthopedics, Deformity Reconstruction and Foot Surgery, University Hospital Muenster, 48149 Muenster, Germany; maike.niemann@ukmuenster.de (M.N.); gregor.toporowski@ukmuenster.de (G.T.); andrea.laufer@ukmuenster.de (A.L.); robert.roedl@ukmuenster.de (R.R.); bjoern.vogt@ukmuenster.de (B.V.); 2 General Orthopedics and Tumor Orthopedics, University Hospital Muenster, 48149 Muenster, Germany; georg.gosheger@ukmuenster.de (G.G.); thomas.ackmann@ukmuenster.de (T.A.); 3 Institute of Biostatistics and Clinical Research, University of Muenster, 48149 Muenster, Germany; maria.eveslage@ukmuenster.de

**Keywords:** leg length discrepancy, tibial epiphysiodesis, fibular epiphysiodesis, dPTFH, proximal fibula, children

## Abstract

The need for concomitant proximal fibular epiphysiodesis (PFE) when correcting leg length discrepancy (LLD) with temporary proximal tibial epiphysiodesis (PTE) in children is controversially discussed. This single center, retrospective cohort study analyzes proximal fibular growth in patients treated by PTE with and without concomitant PFE. Radiographic measurements were conducted before implantation and at implant removal. The position of the fibular head in relation to the tibia was assessed with recently established radiographic reference values. All patients (*n* = 58, 19 females) received PTE to treat LLD at a mean age of 12.2 years (range 7 to 15). In 27/58 (47%) concomitant PFE was performed. Mean follow-up was 36.2 months (range 14.2 to 78.0). The position of the proximal fibula at implant removal was within physiological range in 21/26 patients (81%) with PFE and in 21/30 patients (70%) without PFE. Proximal fibular overgrowth newly developed in 2/26 patients (8%) treated with PFE and in 5/30 patients (17%) treated without PFE (*p* = 0.431). Peroneal nerve injury or discomfort due to proximal fibular overlength was not reported. The position of the proximal fibula should be critically assessed preoperatively under consideration of reference values before PTE. In consequence of this study, the authors do not routinely perform PFE concomitantly with PTE for correction of moderate LLD in children if the proximal fibula is localized within physiological radiographic margins determined by the established reference values.

## 1. Introduction

Growth arrest by epiphysiodesis is a common surgical procedure to correct moderate leg length discrepancy (LLD) in children [1,2,3]. The intervention is usually performed at the growth plates of the distal femur and proximal tibia [1,2,3,4,5,6]. Permanent techniques include ablation of the growth plate in a percutaneous or open manner [1,6]. The application of screws, staples, or plates bridging the physis represent common techniques for temporary growth arrest [3,7,8]. Low complication rates have been described for epiphysiodesis being less invasive than corrective osteotomies [1,2,3,4,5,6,7,8,9,10]. To date there is no clear consensus if proximal fibular epiphysiodesis (PFE) should be performed concomitantly with proximal tibial epiphysiodesis (PTE). 

To prevent fibular overgrowth and instability of the lateral collateral ligament (LCL) some surgeons argue in favor of concomitant PFE with PTE [8,11,12]. Others estimate that the amount of potential fibular overgrowth is clinically inconsequential and recommend not to perform PFE due to the risk of peroneal nerve injury [5,9,13,14,15]. Part of this controversy was sustained by the lack of radiographic reference values defining the physiological proximal tibiofibular relation. Recently a new standard radiographic reference for proximal fibular height in children has been described [16]. The goal of this study is to assess the need and effect of PFE when performing PTE and to analyze radiographic changes of the proximal tibiofibular relation considering specific reference values [16]. 

## 2. Materials and Methods

### 2.1. Patients

**Figure 1:**
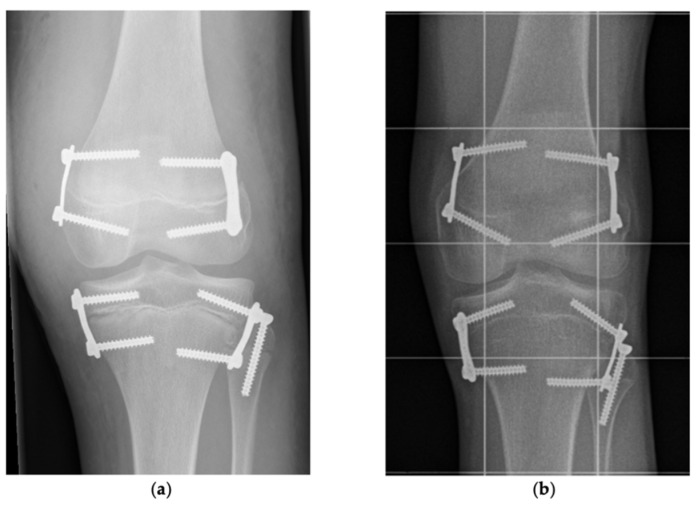
Proximal tibial epiphysiodesis with proximal fibular epiphysiodesis. (**a**) After implantation: Anteroposterior radiograph of a left knee showing proximal tibial epiphysiodesis with PediPlates^TM^ and proximal fibular epiphysiodesis with 36 mm fully threaded cannulated screw in 15-year-old boy with lateral collateral ligament (LLD) of 3.3 cm. (**b**) Before implant removal: Same patient after a treatment time of 36.5 month. The fibular head is localized more distal than the center of the proximal tibial growth plate indicating fibula shortening (dPTFH = −14.5 mm). dPTFH = Distance between the center of the proximal tibial growth plate and a line tangential to the tip of the fibular head horizontal to the imaging plane.

**Figure 2:**
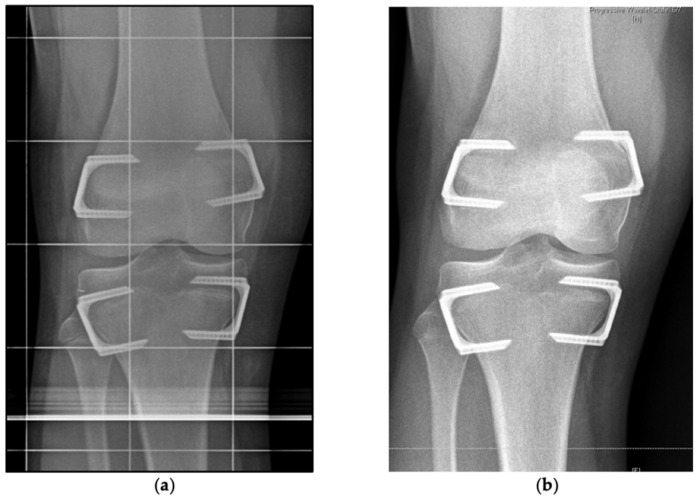
Proximal tibial epiphysiodesis without proximal fibular epiphysiodesis. (**a**) After implantation: Anteroposterior radiograph showing proximal tibial epiphysiodesis without proximal fibular epiphysiodesis on a right knee performed with RigidTacks^TM^ in a 14-year-old boy with LLD of 2.3 cm. (**b**) Before implant removal: Same patient after a treatment time of 19.6 month with proximalization of the fibular head right before implant removal. The fibular head is localized higher than the center of the proximal tibial growth plate (dPTFH = 1.9 mm).

All patients treated with temporary epiphysiodesis for correction of LLD between 2009 and 2020 at our department were retrospectively analyzed. Only patients who received temporary PTE either with or without concomitant PFE at an age between 7 and 16 years for correction of a predicted LLD between 2 and 5 cm at maturity were included (Figure 1 and Figure 2). Patients with fibular hemimelia and congenital short femur were included if the unaffected leg was treated. Patients with permanent epiphysiodesis, history of previous surgery of the longer leg or a mechanical axis deviation (MAD) ≥ 2.5 cm, as well as patients who received systemic growth-affecting treatment such as hormone- or chemotherapy were excluded.

### 2.2. Indication, Operative Technique, and Applied Implants

**Figure 3:**
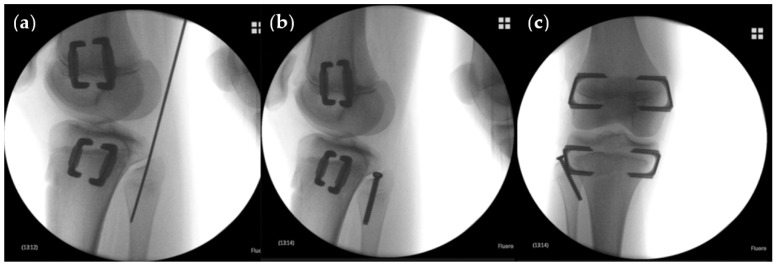
Operative technique of proximal fibular epiphysiodesis. (**a**) Intraoperative lateral radiograph of proximal fibular epiphysiodesis with a cannulated fully threaded screw implanted in a K-wire guided technique concomitantly with distal femoral and proximal tibial temporary epiphysiodesis in a 12-year-old boy. (**b**) Intraoperative lateral radiograph of same patient after implantation. (**c**) Intraoperative anteroposterior radiograph of same patient after implantation.

In children with sufficient residual growth and a predicted LLD between 2 and 5 cm distal femoral epiphysiodesis and PTE was considered to equalize or reduce LLD. Referring to previous recommendations [1,11], PFE was conducted if the remaining tibial growth was estimated greater than 1.5–2.5 cm at the surgeon’s discretion. Surgical treatment of LLD ≥ 5 cm consisted of lengthening by distraction osteogenesis either using external fixators or intramedullary lengthening nails. For conservative treatment of LLD insoles, shoe lifts, and orthotic fittings were applied. After application of intravenous antibiotic (Cefuroxime) the patients were placed on a radiolucent table with a tourniquet on thigh level. The tibial growth plate was fluoroscopically localized and the implants were inserted medially and laterally through minimal invasive approaches preserving the periosteum. For concomitant PFE a cannulated screw was implanted in a K-wire guided technique from the proximal lateral towards the distal medial aspect of the fibula head (Figure 3).

Patients were routinely followed every six months by clinical and radiographic examination. After equalization of LLD or closing of the growth plates implants were removed (mean time of treatment: 26.5 months; range 8.4 to 77.9). After implantation and removal surgery immediate full weight bearing was permitted.

### 2.3. Implants Applied for Epiphysiodesis

PTE was performed with non-locking two hole plates (eight-Plate^TM^, Orthofix, Verona, Italy and PediPlate^TM^, OrthoPediatrics, Warsaw, IN, USA) or staples (RigidTack^TM^ and FlexTack^TM^, Merete Medical GmbH, Berlin, Germany). PFE was conducted with fully threaded cannulated screws (Orthofix and OrthoPediatrics) (Table 1 and Table 2).

### 2.4. Clinical Analysis

Clinical information was acquired from the hospital records focusing on the assessment of knee instability, potential discomfort by proximal fibular overgrowth, and peroneal nerve injury.

### 2.5. Radiographic Analysis

**Figure 4:**
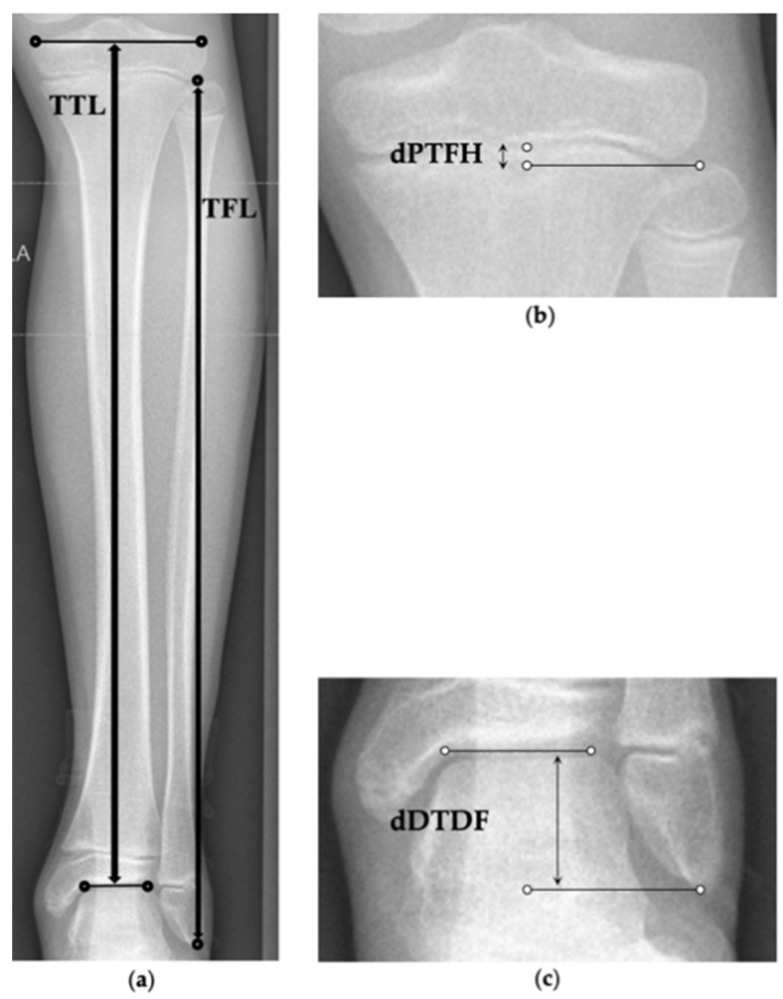
Radiographic measurement parameters. (**a**) TTL = Total tibial length measured from the joint line of the proximal to distal tibia (cm). TFL = Total fibular length measured from the tip of the proximal to the distal fibula (cm). (**b**) dPTFH = Distance between the center of the proximal tibial growth plate and a line tangential to the tip of the fibular head horizontal to the imaging plane (mm) [16]. (**c**) dDTDF = Distance between the articular surface of the distal tibia and a line horizontal to the imaging plane tangential to the distal tip of the fibula (cm) [18].

On calibrated long standing anteroposterior radiographs from the archives of our orthopaedic clinic measurements were conducted with the Picture Archiving and Communication System (PACS, GE Healthcare, Chicago, IL, USA) preoperatively before implantation and at implant removal. The following parameters were analyzed: LLD, predicted LLD determined using the Multiplier Method [17] for congenital etiologies and the Straight Line Graph [18] for developmental or acquired etiologies, total tibial length (TTL), total fibular length (TFL), joint line convergence angle (JLCA), and MAD. The tibia-fibula ratio (TFR) was calculated by dividing TTL by TFL. According to the recently introduced reference values [16] the position of the proximal fibula was assessed by measuring the distance between the center of the proximal tibial growth plate and a line tangential to the tip of the fibular head and horizontal to the imaging plane (dPTFH). The relation of the distal tibia and distal fibula (dDTDF) was measured, as previously described [19] (Figure 4). Secondary angular deformities were defined as treatment related MAD change of ≥15 mm.

### 2.6. Statistical Report

Descriptive statistics were performed using means with ranges (minimum and maximum) for continuous variables and numbers and percentages for binary variables. Distribution was assessed by Shapiro–Wilk test. Mean values were compared with the paired or unpaired Student’s *t*-test, Mann–Whitney–*U* test or signed-rank Wilcoxon test. Analyses adjusting for predicted LLD were performed using multivariable linear regression. Results are reported as regression coefficients with corresponding 95% confidence intervals and *p*-values. Dichotomous variables were analyzed by the exact Fisher test or the exact McNemar test. The local level of significance was set at α < 0.05. All statistical tests were conducted using SPSS 26 (IBM, Armonk, NY, USA).

## 3. Results

### 3.1. Patient Characteristics and Surgical Parameters

In total, 58 patients (19 females) who met the inclusion criteria were identified. The mean chronological age at surgery was 12.2 years (range 7 to 15; mean age females: 11.7 years, range 9 to 14; males: 12.5 years, range 7 to 15) (Table 3). The group treated with PFE was younger than the cohort treated without PFE (mean 11.6 years vs. 12.7 years, *p* = 0.037). The most common etiologies were idiopathic, hemihypertrophic and posttraumatic LLD (Table 4). 

All patients (100%) received PTE and in 51 of 58 patients (88%) temporary epiphysiodesis of the distal femur was performed simultaneously. PFE was conducted concomitantly with PTE in 27 of 58 operations (47%) (Table 3). Long standing anteroposterior radiographs were available from all patients (100%) before implantation and from 56 out of 58 patients (97%) at implant removal. One patient (2%) underwent implant removal in another hospital, another patient (2%) was lost to follow-up. To date implants were routinely removed after the end of treatment in 55 of 58 patients (95%). The mean time to implant removal was 29.6 months (range 9.7 to 78.0). The mean age at implant removal was 14.7 years (range 9 to 18; mean age females: 14.3 years, range 12 to 17; males: 14.9 years, range 9 to 18). The mean follow-up period was 36.2 months (range 14 to 78). 

The mean LLD of the entire cohort decreased from 2.8 cm (2–5) preoperatively to 1.4 cm (0–4) at implant removal (*p* < 0.001). Before beginning of treatment the mean predicted LLD was 3.2 cm (range 2 to 5) (Table 5). 

Secondary angular deformities due to unevenly distributed growth arrest of the distal femur or proximal tibia were observed in 8/58 patients (14%). 4/27 patients (15%) were treated with PFE and 4/31 patients (13%) without PFE. Until skeletal maturity physiological limb alignment was restored in all of these patients by means of temporary hemi-epiphysiodesis through premature removal of the concave-sided implants.

### 3.2. Radiographic Outcome Regarding Tibiofibular Relation

#### 3.2.1. Comparison in Each of the Groups Treated with and without PFE 

During treatment, statistically significant changes of the mean TFR and dPTFH were observed in patients treated without PFE in contrast to the patients treated with PFE. No statistically significant difference of the JLCA before implantation and at implant removal was found in each group. From implantation to removal a statistically significant change of the MAD was detected in the group treated without PFE in contrast to the group treated with PFE. These changes were minimal, within physiological margins and considered clinically inconsequential (Table 5).

#### 3.2.2. Comparison between the Groups Treated with and without PFE

**Figure 5:**
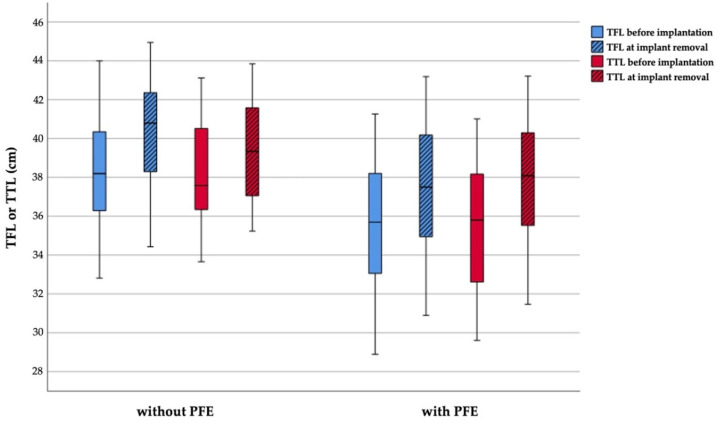
Boxplot graphs depicting the total fibular length (TFL) and total tibial length (TTL) at implantation and before implant removal subdivided by groups treated with and without proximal fibular epiphysiodesis.

Before treatment no statistically significant differences of the mean TFR, dPTFH, and dDTDF were found between the groups (Table 6). This is also confirmed in multivariable analyses, adjusting for predicted LLD (Table 7). At implant removal statistically significant changes of all three parameters were observed between the two groups. Multivariable analyses, adjusting for predicted LLD, show that the change in dPTFH does not differ between patients treated with and without PFE whereas statistical significant changes of the TFR were found (Table 8). Since the observed absolute changes and their statistical significance rely on alterations of only few millimeters further distribution analyses were conducted to assess the proximal tibiofibular relation (Table 9). The lengths of the fibula and tibia before and at the end of treatment are depicted by in Figure 5.

**Figure 6:**
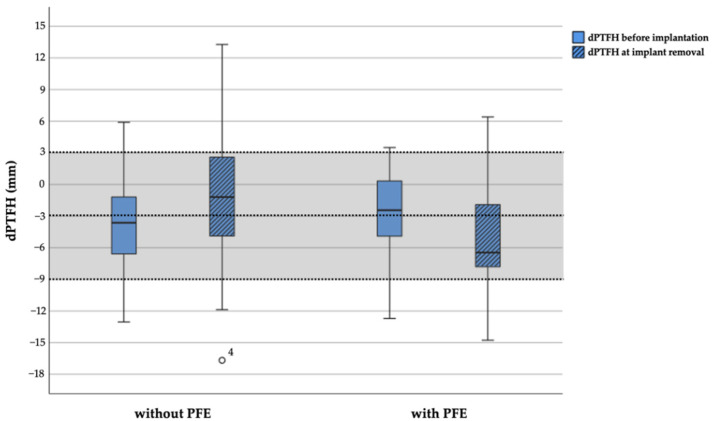
Boxplot graph depicting the distance from the proximal tibial physis to the fibular head (dPTFH) at implantation and before implant removal subdivided by groups treated with and without proximal fibular epiphysiodesis. Dashed lines indicate the physiological mean value of dPTFH (=−3 mm) and two standard deviations (−9 mm, 3 mm) which are defined as the physiological range (light grey area) [16]. One outlier is shown as a circle. PFE = proximal fibular epiphysiodesis.

**Figure 7:**
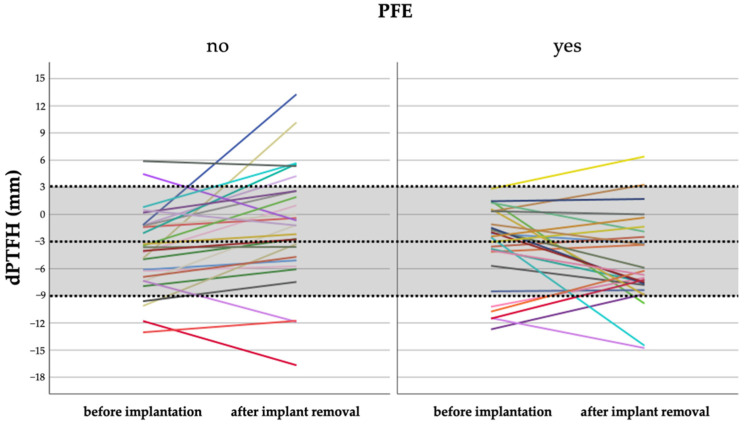
Development of dPTFH form the date of implantation compared to before implant removal. Every line represents one patient. Dashed lines depict the normal mean value of dPTFH (=−3 mm) and two standard deviations (light grey area).

According to the defined physiological range of dPTFH proximal fibular overgrowth was present before treatment in 1 of 27 patients (4%) treated with PFE and in 2 of 31 patients (7%) treated without PFE. After treatment fibular overgrowth was recorded in 2 of 26 patients (8%) and fibular shortening in 3 of 26 patients (12%) treated with PFE, whereas fibular overgrowth was present in 6 of 30 patients (20%) and fibular shortening in 3 of 30 (10%) patients without PFE. At implant removal the mean dPTFH remained within its physiological range in 21 of 26 patients (81%) with PFE and in 21 of 30 patients without PFE (70%) (Figure 6 and Figure 7). Fibular overgrowth newly developed in 2 of 26 patients (8%) with PFE, and 5 of 30 patients (17%) treated without PFE (*p* = 0.431) (Table 9, Figure 5).

### 3.3. Clinical Outcome

**Figure 8:**
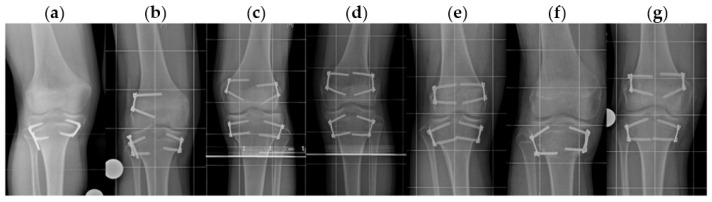
Anteroposterior radiographs of the 7 patients in whom newly occurred proximal fibular overgrowth was observed at the end of treatment (**a**,**b**) with (*n* = 2) and (**c**–**g**) without temporary proximal fibular epiphysiodesis (*n* = 5).

A total of 10 of the 58 patients (17%) reported moderate pain postoperatively at the operation site during ambulation. In 7 patients, pain resolved spontaneously without analgesic treatment. Overall, 3 patients required oral non-steroidal anti-inflammatory drugs until pain relief. No injury or sensorimotor dysfunction related to the peroneal nerve was observed in patients treated with PFE. No discomfort due to fibular overgrowth or knee joint instability due to potential laxity of the LCL was found in the cohort treated without PFE. Especially, discomfort due to fibular overgrowth, clinical laxity of the knee, restrictions of daily activity or recreational sports were not reported by the 7 patients who newly developed fibular overgrowth (Figure 8).

## 4. Discussion

Temporary or permanent epiphysiodesis are established methods to treat LLD from 2–5 cm in children by arresting distal femoral and proximal tibial growth [2,4,9,10,12,20]. To date, temporary epiphysiodesis is commonly and successfully performed [2] with different types of implants such as transphyseal screws [8], screw-plate-devices or staples bridging the growth plate [21,22,23].

Irrespective of the applied technique for epiphysiodesis, surgeons must decide preoperatively if concomitant PFE is necessary when performing PTE. There is a lack of studies focusing on this specific question.

Some surgeons avoid performing PFE due to the risk of peroneal nerve injury and expect the resulting fibular overgrowth to be clinically irrelevant [5,9,13,15]. Several studies were published describing the outcome of PTE without concomitant PFE. Siedhoff et al. published a series of 34 children treated by temporary PTE without PFE [9]. Gabriel et al. analyzed the outcome of 29 patients treated with permanent PTE of whom 16 did not receive concomitant PFE [13]. Both studies found no clinical consequences for the patients treated without PFE [9,13]. Several other authors also refrain from concomitant PFE such as Green and Anderson [24,25] or Makarov et al., who evaluated the complications of permanent epiphysiodesis for correction of LLD in a series of 836 patients of whom 508 were treated by femoral with tibial or just tibial arrest. Unfortunately, the number of patients who received concomitant PFE with PTE was not provided. Surgical injury of the peroneal nerve was observed in only one patient (0.2%) [10]. In 1 of 12 patients (8%) Canale et al. reported persistent peroneal nerve palsy after permanent percutaneous PFE and concluded that PFE is usually not necessary if the desired amount of proximal tibial growth arrest is less than 2.5 cm [1]. This study did not observe an increased risk of complications such as peroneal nerve injury, functional discomfort, or knee instability when performing temporary PTE with or without concomitant PFE. 

Recently, Boyle et al. analyzed 243 patients treated by PTE for LLD and concluded that PFE should only be performed in patients with preoperative proximal fibular prominence or at least 2 years of remaining growth [14]. However, proximal fibular prominence is not clearly defined and appears to be the subjective rating of the authors. 

Other surgeons argue in favor of PFE to prevent fibular overgrowth and potential instability of the LCL [8,11,12,26]. Metaizeau et al. suggest PFE after observing progressive tibia varus malalignment in three patients with continued growth of the non-treated fibula when desired tibial correction exceeded 2 cm [8]. Porat et al. reported no peroneal nerve damage or fibular overgrowth in their series of 20 children who received PFE concomitantly to permanent PTE [12]. McCarthy et al. stated that PFE is a safe procedure and should be considered in patients with predisposing or expected fibular overgrowth of more than 1–2 cm. Fibular overgrowth was calculated as the difference of the distance from the proximal tibial growth plate to the fibular head measured preoperatively and at skeletal maturity [11]. 

This is the first study which analyzes the proximal tibiofibular relation under consideration of dPTFH as the only available radiographic reference value. dPTFH was recently introduced defining the physiological position of the proximal fibula in children aged between 8 and 16 years aiming to aid preoperative decision-making based on objective data when performing epiphysiodesis [16]. The use of dPTFH for measurements of fibular overgrowth is a validated and standardized radiographic approach in contrast to McCarthy et al. [11]. 

In the studied cohort the position of the proximal fibula at implant removal was found to be within physiological range in most of the patients treated with PFE and without PFE. The incidences of newly developed proximal fibular overgrowth were low and only observed in 8% of patients treated with PFE and 17% of patients treated without PFE. On the one hand these findings show that treatment without PFE can lead to radiographically measured proximal fibular overgrowth in up to one of six procedures. On the other hand the results demonstrate that in accordance with observations made by Boyle et al. [14] PFE does not unequivocally prevent fibular head overgrowth. 

In accordance to Boyle et al. [14], statistically significant changes of the TFR were detected during treatment in the cohort treated without PFE. To the knowledge of the authors normal and age-dependent reference values of the TFR do not exist to further classify and evaluate this observation. The authors believe that the statistically significant differences of the TFR between patients with and without PFE are clinically irrelevant. 

In consistence with the observations of McCarthy et al. and Boyle et al. [11,14] a statistically significant overgrowth of the distal fibula in the patients treated without PFE was observed in this study. Based on the observations by Pritchett et al. this finding is most likely explained by the natural history of fibular growth. The fibula descents on the tibia due to an unevenly distributed tibial and fibular appositional growth potential and pulling talofibular ligaments [27].

All angular deformities identified in the study cohort were related to alterations of the mechanical knee joint alignment angles representing a common problem of temporary epiphysiodesis for LLD correction [2,21]. In none of these patients a treatment related pathological alteration of the position of the proximal fibula was observed. These findings are supported by observations from Siedhoff et al. who found no relevant MAD alteration analyzing the outcome of 34 patients treated with temporary PTE without concomitant PFE [9]. The mean values of JLCA did not show statistically or clinically significant differences within and between the groups before epiphysiodesis and at implant removal.

To date studies questioning the need for concomitant PFE when treating children with PTE for LLD are underrepresented since only two previous studies have investigated this specific issue [11,14]. Recent studies did not comment on the existing controversy and presented results without explicitly indicating if PFE was concomitantly conducted or not. Moreover, proximal fibular shortening after PFE and its clinical consequences have never been discussed yet. One could argue that similar to fibular overgrowth, fibular shortening during growth could result in tethering of the LCL eventually leading to a valgus knee alignment or medial instability. In this study we could not observe any instability or secondary angular deformities related to fibular overgrowth or shortening. 

The clinical consequences of proximal fibular overgrowth are still insufficiently described in literature and have hindered definite recommendations if and when PFE should be performed concomitant to PTE. Even though new reference values help the radiographic assessment of the proximal fibula [16] there is still no threshold that defines when proximal fibular overgrowth becomes functionally impairing. Furthermore a specific conclusion and comparison remains challenging since previous studies lack to describe if their results were obtained from permanent epiphysiodesis solely or if temporary epiphysiodesis was included as well [11,14].

So far the findings of the longitudinal radiographic study evaluating fibular growth in children by Pritchett et al. have found little attention in the debate. The study has demonstrated that the proximal fibula grows 1 cm per year in adolescents [27]. For example, assuming this growth rate after PTE in a 12-year-old male without PFE would result in approximately 4 cm of proximal fibular growth. This would commonly lead to a localization of the fibular head proximal to the knee joint with expectable severe disfunction of the proximal tibiofibular joint and lateral knee instability. However, this was never observed in this study nor a common finding of previous studies [11,14]. The authors hypothesize that similar to the ankle [27] the strong ligamentous complex of the proximal tibiofibular joint prevents the development of proximal fibular overgrowth at its physiological rate and that the effect of PFE might play a subordinate or even neglectable role.

This study has several limitations due to its retrospective design. Ideally, a prospective and randomized or matched pair study is required to reliably assess the influence of temporary PTE for LLD treatment with or without PFE. Patient reported outcome measures, such as pain and treatment influence on daily activity, are insufficiently reported. Standardized and repetitive physical examinations of ligamentous stability are needed to assess the clinical influence of minimal radiographic changes of the position of the proximal fibula which this study cannot provide. Another potential source of bias is the uneven distribution of etiologies and the use of different types of implants. Only patients treated with temporary epiphysiodesis were analyzed, so that not the same conclusions might be applicable for patients with permanent epiphysiodesis. It is important to consider that in 88% of the patients the amount of LLD correction was achieved by combined distal femoral and proximal tibial arrest.

## 5. Conclusions

The analyzed patient cohort with a mean age of 12 years and mean preoperative LLD of 3 cm is representative for the typical spectrum of indication for treatment of LLD in children by means of epiphysiodesis. 

New radiographic reference values such as dPTFH should be implemented in daily clinical routine to detect shortening or overgrowth of the proximal fibula before treatment.

Even when PTE is conducted without PFE proximal fibular growth is reduced compared to its physiological growth rate.

No distinct radiographic difference of the proximal fibular position was found between the subgroups with or without PFE.

As a consequence of this study the authors do not routinely perform temporary PFE concomitantly with temporary PTE for LLD correction of 2–5 cm in children when the proximal fibula is preoperatively localized within physiological radiographic margins determined by dPTFH. 

## Figures and Tables

**Table 1:** Implants used for proximal tibial epiphysiodesis.

Implant	*n* (58)
eight-Plate^TM^	31
PediPlate^TM^	6
FlexTack^TM^	5
RigidTack^TM^	16

**Table 2:** Cannulated screws used for proximal fibular epiphysiodesis.

Length	*n* (27)
30 mm	2
32 mm	15
36 mm	10

**Table 3:** Patients demographics.

**Number of Patients**	**58**
Male	39 (67%)
Female	19 (33%)
**Mean age in years at surgery (range)**	**12.2 (7–15)**
Male	12.5 (7–15)
Female	11.7 (9–14)
**Mean age in years at implant removal (range)**	**14.7 (9–18)**
Male	14.9 (9–18)
Female	14.3 (12–17)
**Type and number of operations**	**58**
Exclusively proximal tibial epiphysiodesis	7 (12%)
Combined proximal tibial and distal femoral epiphysiodesis	51 (88%)
Concomitant proximal fibular epiphysiodesis	27 (47%)

**Table 4:** Etiologies of the patient cohort.

Etiology	*n*
*Congenital LLD*	
Fibular hemimelia	5
*Developmental or acquired LLD*	
Idiopathic	17
Hemihypertrophy	16
Posttraumatic	6
Hip pathology	6
Club foot	4
Neuromuscular	2
Post-infectious	1
Trisomy X	1

LLD = leg length discrepancy.

**Table 5:** Comparison of mean values and ranges of the assessed radiographic parameters before implantation and at implant removal in the patient groups treated with and without concomitant proximal fibular epiphysiodesis (PFE). The *p*-values in the last column are the statistical difference between the mean differences (MD) of the two groups (ΔMD).

	Group Treated with PFE (*n* = 27)				Group Treated without PFE (*n* = 31)				ΔMD
	Before Implantation	At Implant Removal	*p*-Value	MD	Before Implantation	At Implant Removal	*p*-Value	MD	*p*-Value
Age (years)	11.6 (7–15)	13.5 (8–18)	/	2.3 (1–5)	12.7 (9–15)	15 (12–18)	/	2.5 (1–6)	/
LLD (cm)	3.2 (2–5)	1.5 (0–4)	<0.001	1.8 (0.2–3.5)	2.4 (2–5)	1.2 (0–4)	<0.001	1.3 (0.2–3.4)	0.062
Predicted LLD (cm)	3.8 (2–5)	/	/	/	2.7 (2–5)	/	/	/	/
MAD (mm)	−1 (−15–11)	−3 (−27–29)	0.234	8.3 (0–27)	−2 (−17–11)	3 (−17–27)	0.006	8.2 (1–29)	0.837
JLCA (°)	1.2 (0–2.6)	1.2 (0–4.5)	0.841	1.1 (0–3.2)	1.2 (0.1–5.0)	1.1 (0–4.1)	0.436	0.8 (0–4.1)	0.185
Total tibial length (cm)	35.6 (29.6–41.0)	37.6 (31.5–43.2)	<0.001	2.0 (0.2–5.3)	38.3 (33.7–43.1)	39.6 (35.2–43.9)	<0.001	1.8 (0.1–5.4)	0.755
Total fibular length (cm)	35.6 (28.9–41.3)	37.5 (30.9–43.2)	<0.001	2.1 (0.2–5.7)	38.5 (32.8–44.0)	40.5 (34.4–45.0)	<0.001	2.3 (0.1–6.3)	0.819
Tibia-Fibula ratio	1.00 (0.96–1.09)	1.00 (0.96–1.05)	0.427	0 (0–0.1)	0.99 (0.95–1.04)	0.98 (0.95–1.02)	<0.001	0 (0–0.1)	0.250
Distance proximal tibial physis to fibular head (mm; dPTFH) [16]	−3.4 (−12.7–3.5)	−5.1 (−14.8–6.4)	0.223	3.7 (0.1–12)	−3.7 (−13.0–5.9)	−1.2 (−16.7–13.3)	0.005	3.9 (0–15)	0.876
dPTFH within physiological range (%)	21/27 (78)	21/26 (81)	1.000	/	25/31 (81)	21/ 30 (70)	0.508	/	/
Distal tibio-fibular distance (cm; dDTDF) [18]	2.2 (1.4–2.9)	2.1 (0.8–3.0)	0.171	0.2 (0–0.6)	2.3 (1.5–3.5)	2.5 (1.8–3.4)	<0.001	0.3 (0–0.6)	0.335

MAD = mechanical axis deviation; JLCA = joint line convergence angle.

**Table 6:** Mean values and ranges of the assessed radiographic parameters are compared between the groups treated with PFE and without PFE before epiphysiodesis and at implant removal.

	Before Implantation			At Implant Removal		
	With PFE	Without PFE	*p*-Value	With PFE	Without PFE	*p*-Value
Tibia-Fibula ratio	1.00 (0.96–1.09)	0.99 (0.95–1.04)	0.798	1.00 (0.96–1.05)	0.98 (0.95–1.2)	<0.001
Distance proximal tibial physis to fibular head (dPTFH; mm) [16]	−3.4 (−12.7–3.5)	−3.7 (−13.0–5.9)	0.546	−5.1 (−14.8–6.4)	−1.2 (−16.7–13.3)	0.018
dPTFH within physiological range (%)	77.8	80.6	1.000	80.8	70.0	0.508
Distal tibio-fibular distance (dDTDF; cm) [18]	2.2 (1.4–2.9)	2.3 (1.5–3.5)	0.236	2.1 (0.8–3.4)	2.5 (1.8–3.4)	<0.001

**Table 7:** Results of multivariable linear regression analyses for dPTFH before implantation, at implant removal, and the change between implantation and removal. RC = Regression coefficient, CI = Confidence interval.

	dPTFH (mm)								
	Before Implantation			At Implant Removal			Change		
	RC	95% CI	*p*-Value	RC	95% CI	*p*-Value	RC	95% CI	*p*-Value
Intercept	−4.2	(−5.7; −2.7)	<0.001	−1.9	(−4.1; 0.2)	0.077	4.0	(2.7; 5.3)	<0.001
PFE (yes vs. no)	1.4	(−0.9; 3.7)	0.234	−2.5	(−5.7; 0.8)	0.142	−0.4	(−2.4; 1.6)	0.677
Predicted LLD (cm) centered at mean	−1.5	(−2.8; −0.3)	0.012	−1.7	(−3.5; −0.01)	0.048	0.2	(−0.8; 1.3)	0.677

**Table 8:** Results of multivariable linear regression analyses for the Tibia-Fibula ratio before implantation, at implant removal, and the change between implantation and removal. RC = Regression coefficient, CI = Confidence interval.

	Tibia-Fibula Ratio								
	Before Implantation			At Implant Removal			Change		
	RC	95% CI	*p*-Value	RC	95% CI	*p*-Value	RC	95% CI	*p*-Value
Intercept	0.997	(0.989; 1.005)	<0.001	0.981	(0.974; 0.988)	<0.001	−0.016	(−0.022; −0.010)	<0.001
PFE (yes vs. no)	−0.002	(−0.014; 0.009)	0.678	0.019	(0.008; 0.029)	0.001	0.019	(0.010; 0.028)	<0.001
Predicted LLD (cm) centered at mean	0.008	(0.002; 0.014)	0.010	0.005	(−0.001; 0.011)	0.083	−0.002	(−0.007; 0.002)	0.332

**Table 9:** Assessment of proximal fibular overgrowth and shortening during treatment.

	Group Treated with PFE (*n* = 27)			Group Treated without PFE (*n* = 31)		
	Before Implantation	At Implant Removal	*p*-Value	Before Implantation	At Implant Removal	*p*-Value
dPTFH out of physiological range [16]	6/27 (22%)	5/26 (19%)	1.000	6/31 (19%)	9/30 (30%)	0.508
Proximal fibular overgrowth	1/27 (4%)	2/26 (8%)	0.500	2/31 (6%)	6/30 (20%)	0.219
Newly developed overgrowth	/	2/26 (8%)	/	/	5/30 (17%)	/
Proximal fibular shortening	5/27 (19%)	3/26 (12%)	0.687	4/31 (13%)	3/30 (10%)	1.000
Newly developed shortening	/	2/26 (8%)	/	/	1 (3%)	/

## Data Availability

The datasets generated during or analyzed during the current study are available from the corresponding author on reasonable request.
